# Ergogenic and Sympathomimetic Effects of Yohimbine: A Review

**DOI:** 10.3390/neurolint16060131

**Published:** 2024-12-12

**Authors:** Sophia L. Porrill, Rebecca R. Rogers, Christopher G. Ballmann

**Affiliations:** 1 Department of Human Studies, University of Alabama at Birmingham, Birmingham, AL 35294, USA; sporrill@uab.edu; 2 SHP Research Collaborative, University of Alabama at Birmingham, Birmingham, AL 35294, USA; 3 Department of Family and Community Medicine, University of Alabama at Birmingham, Birmingham, AL 35294, USA; rrrogers@uab.edu; 4 Department of Physical Therapy, University of Alabama at Birmingham, Birmingham, AL 35294, USA

**Keywords:** catecholamines, dietary supplement, sympathetic nervous system, stimulant

## Abstract

The purpose of this review is to compile and discuss available evidence in humans on the efficacy of YHM supplementation on performance in different exercise modalities. Yohimbine (YHM) is a naturally occurring alkaloid that induces increases in sympathetic nervous system (SNS) activation effectively initiating “fight or flight” responses. In supplement form, YHM is commonly sold as an isolated product or combined into multi-ingredient exercise supplements and is widely consumed in fitness settings despite the lack of empirical support until recently. YHM primarily acts as an α2-adrenergic receptor antagonist effectively increasing norepinephrine release from sympathetic neurons. YHM has been implicated in improving or altering cardiovascular function, blood flow, lactate metabolism, and muscle function. Emerging evidence has suggested that YHM may have the potential to improve performance in a wide range of exercise modes including endurance, sprint, and resistance exercise. Performance enhancement with YHM is mediated by mechanistic underpinnings of physiological and psychological alterations to exercise responses including increased sympathetic activation, adaptive hemodynamic changes, increased alertness, and decreased fatigue. However, YHM use is not without risk as it has high interindividual variability in bioavailability, can be deceptively potent, lacks widely accepted dosing recommendations, and, when taken in large doses, has been empirically documented to result in serious side effects. Despite this, the evidence presented in this review suggests low doses of YHM are tolerable and may serve as an ideal exercise training aid due to acute enhancement of physical performance. However, safety concerns remain outstanding and temperance should be used when using YHM and similar sympathomimetics.

## 1. Introduction

Yohimbine (YHM) is an alkaloid compound derived from the bark of the Corynanthe yohimbe tree native to West Africa [1]. YHM has traditionally been used in African folk and Western medicine to increase feelings of energy and vitality in both animals and humans. Empirically, YHM has been widely studied as a means to improve peripheral circulation in the context of erectile dysfunction and induction of lipolysis/weight loss [2,3,4,5]. YHM is available in ground extract forms from bark or in purified forms. Due to the inconsistencies in concentrations of YHM found in the bark itself, YHM is commonly ingested in its commercially available form of Yohimbine hydrochloride [6]. YHM has been characterized as an α2-adrenergic receptor (AR2) antagonist, which leads to increases in sympathetic nervous system (SNS) activity through increases in circulating catecholamines [1]. Since AR2s serve as regulatory negative feedback to prevent excessive release of norepinephrine (NE), antagonistic actions of YHM lead to systemic increases in sympathetic activity that are underpinned by NE spillover.

The systemic effects of YHM influence multiple organ systems including cardiovascular, neuromuscular, endocrine, and nervous systems. The underlying effects of YHM have been studied in humans and animals, albeit the latter makes up the bulk of the available literature. The general implications of using YHM on the cardiovascular system are the increase in systolic blood pressure and increased blood flow [7], which when paired with the nervous system’s increase in SNS stimulation, can lead to implications in exercise performance and feelings of alertness [8]. Due to the NE spillover phenomenon, which is a trait of YHM ingestion, there is a dramatic increase in circulating NE and epinephrine [9,10,11], which can enhance neuromuscular control and alter fuel utilization for exercise performance [12]. Much of the preliminary physiological effects of YHM have been described decades ago; however, the influence of YHM ingestion on exercise performance is minimal and has only recently been described.

Commercially, YHM is used in multi-ingredient exercise performance supplements despite minimal research on health and performance implications [6]. The adverse effects of YHM at low doses are relatively mild and include temporary hypertension and tachycardia; however, there have been few reported cases of serious adverse effects in sport and exercise when the safety dosage is dramatically exceeded [13]. Although optimal dosing strategies have not been established, many studies recommend the effective oral dose of YHM as 2.5–5 mg [1,8,14,15]. However, multiple doses have been empirically tested, ranging from 2.5 to 20 mg, making definitive dosage recommendations elusive at this time. YHM has been implicated in improved exercise performance both in aerobic and anaerobic modalities [8,14,15,16]. Physiologically, YHM has been shown to increase catecholamines, oxygen consumption, muscular force/power, markers of ATP breakdown, heart rate, and lower lactate levels in response to exercise [1,14,15,16]. The psychological effects of YHM have also been characterized during exercise, revealing increases in motivation, feelings of energy, and alertness/arousal [8,14,17]. The main aim of this review is to gather and analyze the latest available evidence in humans on YHM supplementation and its ergogenic effects on performance across various exercise types. It will also explore the primary physiological and psychological mechanisms through which YHM may influence exercise performance. Additionally, the effectiveness, dosage, safety concerns, and potential clinical translation will be examined, with recommendations provided for coaches, athletes, and practitioners.

## 2. Primary Mechanism of Action

**Figure 1:**
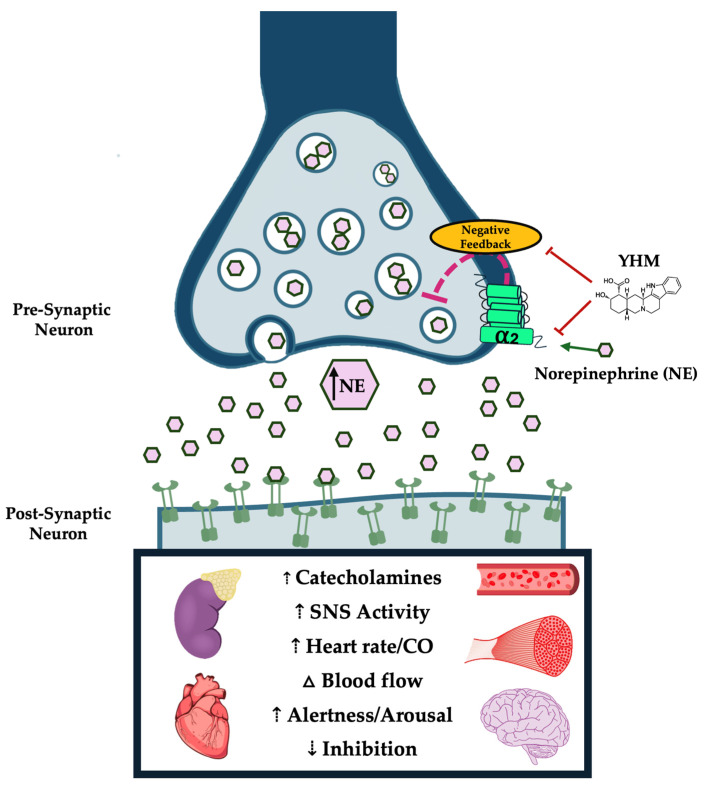
Primary mechanism of action of Yohimbine (YHM) via α_2_-adrenergic receptor antagonism. YHM competes for norepinephrine (NE) binding sites on α_2_-adrenergic receptors, which serve as negative feedback regulators of NE release at the pre-synaptic neuron. Inhibition of a_2_-adrenergic receptor activation results in exacerbation of NE release and NE spillover. The phenomenon of NE spillover leads to the propagation of catecholamine release and sympathetic activation systemically. This influences multiple organ systems including the adrenal glands, heart, vasculature, skeletal muscle, and neural activity. Alterations in skeletal muscle performance, cardiovascular function, hemodynamics, and metrics linked to psychological arousal have been implicated as underlying effects mediating the ergogenic effects of YHM.

The full scope of the mechanisms of action of YHM is still being elucidated despite its long history of being studied in the scientific literature. YHM may have off-target effects through agonism and antagonism of various receptors. However, YHM exerts its primary physiological action through selective inhibition of a-adrenergic receptors. More specifically, YHM possesses a strong binding affinity of AR2 with weak to moderate affinity for a_1_-adrenergic receptors [18]. YHM antagonizes AR2s on pre-synaptic neurons effectively obstructing norepinephrine (NE) binding (Figure 1). Antagonization of AR2s results in the disruption of the negative feedback loop, presumably through the accumulation of intraneuronal cyclic adenosine monophosphate (cAMP), which attenuates hyperpolarization of the pre-synaptic neuron [19]. This results in a cascade of signaling and alterations in ion channel activation causing excessive NE release and the phenomenon of NE spillover [11]. Systemic increases in NE then ensue, initiating widespread sympathetic activation with known actions on adrenal-mediated hormone release, cardiovascular responses, skeletal muscle function, and neural activation.

At the adrenal gland, increases in plasma NE mediated by YHM result in increases in epinephrine release [10,20]. This expectedly results in further exacerbation of sympathetic activation and alterations in metabolism. Likely, alterations in cardiac function manifest from these actions as heart rate, cardiac output, and blood pressure become markedly elevated following YHM administration [7,21]. While changes in cardiac function likely underlie hemodynamic changes in YHM, alterations at the vascular level have been suggested as well. YHM results in vasoconstriction of splanchnic vessels to the internal organs resulting in shunting of blood flow to peripheral tissues, namely skeletal muscle [22]. Localized determination of blood flow may also be modulated through nitric oxide (NO), although how this translates to exercise is currently unknown [23]. Purported increases in blood flow to skeletal muscle may thereby improve exercise recovery, namely through the resynthesis of ATP via phosphocreatine, which could prevent neuromuscular fatigue and preserve muscle force output [24,25]. YHM has also been suggested to alter substrate metabolism, primarily through increasing lipolysis, which may be secondary to increasing plasma levels of epinephrine [12,26].

The mechanisms underlying the psychological effects of YHM have been comparatively less studied but appear to be stimulative in nature. YHM has been shown to increase psychological arousal in multiple studies [27,28]. Increases in alertness are commonly reported with YHM ingestion and can be anxiogenic, which appears to be dose-dependent [8,14,29]. Central actions of YHM have been linked to lower inhibition, impulsivity, and a greater prevalence of risk-taking behavior [29]. This is thought to be a result of greater NE release in the CNS, although, peripheral to central crosstalk cannot be eliminated as a possible cause [11,28]. Acute increases in subjective feelings of energy and lower subjective fatigue scores have been observed with YHM ingestion [8,14]. Ratings of motivation have also been reported to acutely increase with YHM [8].

## 3. Pharmacokinetics and Bioavailability

**Figure 2:**
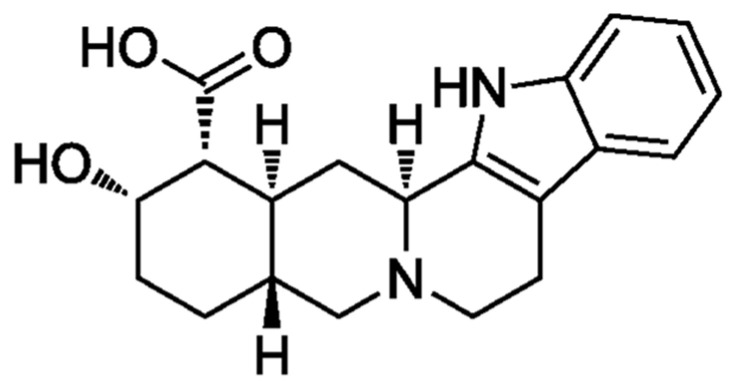
Chemical structure of Yohimbine (YHM). Chemical formula: C_21_H_26_N_2_O_3_. Molecular weight: 354.4 g/mol. Classification: indole alkaloid.

YHM is a naturally occurring nitrogenous compound that is classified as an indole alkaloid and has been characterized repeatedly for its rapid sympathomimetic properties (Figure 2). In dietary form, YHM is quickly absorbed (absorption half-life: 10 min) with plasma levels reaching peak levels 45 min post-ingestion, although this has been shown to have high interindividual variation [25,30]. Documented bioavailability has suggested a highly variable range of 10–90%, likely due to differences in concentration from commercial forms and individual variation in responses [1,30]. YHM is primarily metabolized by the liver and has been shown to be partitioned into red blood cells [22]. The primary action of YHM is through antagonism of α_2_ adrenergic receptor subtypes with high affinity (IC50 = 0.6 μM), although affinity for α_1_ adrenergic, 5-hydroxytryptamine (5-HT), and dopamine receptors have also been reported [25,31]. Complete elimination of YHM is also rapid (elimination half-life: 30–40 min) and occurs primarily through direct metabolic breakdown and hepatic clearance with some renal excretion [22,25,32]. YHM administration has been reported to result in plasma increases in NE from 3- to 5-fold higher than that at rest [32]. Acute oral ingestion of YHM has also been shown to increase epinephrine levels prior to and post-exercise [16].

## 4. Yohimbine and Exercise

**Figure 3:**
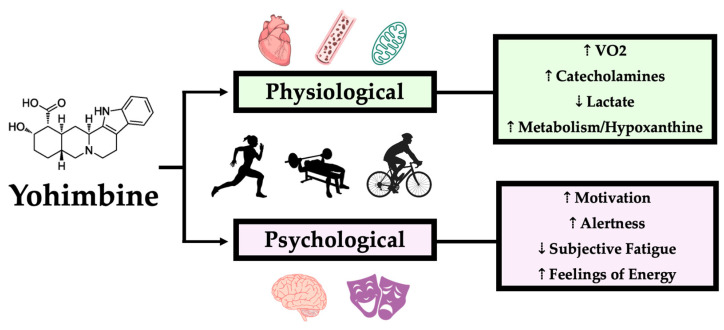
Physiological and psychological factors contributing to improved exercise ability with acute Yohimbine (YHM) ingestion. YHM HCl results in physiological and psychological alterations to mediators of performance. Physiological improvements that have been reported in the literature include greater oxygen uptake (VO2), catecholamine release, and metabolism, as reflected by hypoxanthine. Lower post-exercise lactate levels have also been reported by multiple studies. Psychologically, there have been reports of increased motivation, alertness, and feelings of energy with YHM ingestion. Subjective feelings of fatigue have also been suggested as an underlier to improved performance.

YHM is widely available commercially as a single-ingredient supplement but is more commonly consumed in multi-ingredient supplements as a stimulant additive. While numerous investigations assessing the efficacy of multi-ingredient pre-workout supplements containing YHM have been conducted [33,34,35], the amalgamous ingredient profiles make discerning the distinct contribution of YHM to changes in exercise performance obscure. Thus, this section will solely focus on isolated YHM interventions in the context of exercise performance. In the past two decades, multiple studies using isolated YHM supplementation with the intent of improving exercise performance have been conducted and encompass both aerobic and anaerobic exercise performance (Table 1). The following section will discuss the most recent evidence in humans for YHM ingestion to improve performance in the context of aerobic (e.g., cycling, rowing) and anaerobic (e.g., repeated sprints, bench press) exercise. Furthermore, the underpinning physiological and psychological mechanisms responsible for performance improvement will also be discussed (Figure 3).

**Table 1:** Study table. Listed are the known investigations that have investigated human exercise performance with YHM as an isolated condition including primary findings.

Study	Conditions	Dosage	Ingestion Period	Exercise	Primary Findings
Ostojic et al. (2006) [3]	PL, YHM	20 mg	Daily; 21 days	Sprint (Cycle)	↓ Fat Mass, ↔ Performance
Al-Kuraishy et al. (2014) [15]	PL, YHM	5 mg	2 h	Cycling	↑ VO2, ↑ Distance,↑ Speed, ↑ Caloric Expenditure
Barnes et al. (2022) [17]	PL, YHM	2.5 mg	20 min prior	Sprint (Cycle)	↑ Power, ↑ HR, ↔ RPE, ↑ Epinephrine, ↓ Lactate
Williams et al. (2023) [8]	PL, YHM	5 mg	20 min prior	Bench Press	↔ Power or Velocity, ↑ RTF, ↑ Alertness, ↑ Motivation
Ballmann & Rogers et al. (2024) [14]	PL (AM), YHM (AM), Control (PM)	2.5 mg	20 min prior	Rowing	↑ Power, ↓Time to Completion, ↔ HR or RPE, ↓ Lactate, ↑ Hypoxanthine

Note: VO2 = oxygen consumption, HR = heart rate, RPE = rating of perceived exertion.

### 4.1. Aerobic

Although evidence for the acute effects of YHM ingestion on aerobic exercise is limited, multiple studies have suggested ergogenic benefits [14,15]. In 2014, Al-Kuraishy et al. first described this phenomenon in aerobic cycling [15]. Twenty healthy male medical students participated in a single-blinded crossover design. Participants ingested a single dose of 5 mg of YHM or a placebo (PL) 2 h prior to cycling exercise. Participants cycled as hard and as fast as possible against resistance until volitional fatigue. Outcomes measured included power output (effort), heart rate, distance, time, speed, and estimates of maximal oxygen uptake (VO2max). The authors showed increases in power output, distance covered, average speed, and time to exhaustion with YHM compared to PL. Furthermore, authors also reported increases in resting HR and VO2max with YHM ingestion. The authors attributed the increases in performance to various mechanisms, including hyperexcitability of skeletal muscle, increased cardiac function, and altered fuel consumption during exercise. Although the authors did not measure these metrics directly, there is scientific precedent for their rationale. Indeed, increases in catecholamines have been linked to increases in peak muscle force and may be needed for maintaining optimal force production during exercise [36]. Given the primary mechanism of YHM being related to increases in catecholamines, the improvements in power output and performance metrics may be a result of NE spillover and perpetuation of the stimulatory response. For cardiac function, YHM has been previously shown to increase HR and/or stroke volume (SV) [25,37]. Increases in HR and SV likely lead to greater blood and oxygen delivery to skeletal muscle during exercise, which could explain the increases in VO2max and cycling performance. This is supported by previous evidence showing blood flow to the skin and the brain is altered by YHM, in addition to other reports of increased lactate clearance [7,16,38]. Lastly, YHM and other a2-adrenergic receptor antagonists have been shown to alter fuel utilization, namely by increasing lipolysis and free fatty acid recruitment [39,40]. This could, in turn, result in greater free fatty acid availability, thus increasing efficiency and energy availability during exercise [41].

However, this study presents several limitations, which make drawing conclusions difficult. First, the cycling protocol used was discussed in minimal detail and, as such, it is difficult to discern the nature of the exercise precisely. The most difficult limitation relates to the method by which VO2 was determined. To obtain VO2max, the heart rate ratio method was used as previously described by Uth et al., whereby resting and maximal HR are used to derive relative estimates [42]. While appropriate in certain contexts, the use of the HR ratio to estimate VO2 is likely less accurate than direct measurement since YHM is known to alter HR at rest and during exercise [17,37]. This is bolstered by previous evidence showing that stimulants may alter submaximal and peak exercise cardiac responses to exercise [43,44,45]. However, no study, to date, has directly measured oxygen uptake or fuel utilization leaving potential benefits of YHM to VO2 equivocal.

More recently, Ballmann & Rogers et al. investigated acute YHM ingestion on morning-associated losses in endurance rowing performance [14]. It has been well described that exercise performance tends to suffer during morning (AM) times while peak performance occurs in the afternoon (PM) [46,47]. The authors tested whether YHM ingestion could prevent losses in performance and restore AM performance to peak levels similar to that of PM [48]. Accordingly, a double-blind counterbalanced crossover approach was utilized with the following three different treatments: (1) placebo in the AM (PL), (2) YHM in the AM (YHM), and (3) control (no treatment; PM). Physically active females (n = 16) were recruited for testing, and, for the YHM condition, participants consumed 2.5 mg of YHM 20 min prior to exercise. Participants completed a 2000 m rowing time trial and had blood collected pre- and post-exercise for lactate and hypoxanthine levels. For performance, power output and time to completion were documented. Also, psychometric testing was completed to elucidate feelings of energy, alertness, focus, and fatigue. Results showed that YHM attenuated AM-associated performance decrement (e.g., power output, time) versus PL. Feelings of energy and alertness increased, focus remained unchanged, and fatigue decreased. Post-exercise lactate levels were lower with YHM ingestion while hypoxanthine, an indirect marker of ATP breakdown, trended towards increasing compared to PL. Authors postulated that the increases in power output and improved time to completion may have manifested in alterations of muscle activation. Indeed, YHM possesses sympathomimetic properties, which increase sympathetic output to muscle [25]. The improvements with acute YHM ingestion may manifest in a more rapid induction of sympathetic activity and inotropic effects where muscular force is amplified concomitant to hastened relaxation, which could allow for more frequent and forceful contractions [49,50]. Interestingly, blood lactate was lower despite trends in increases in hypoxanthine. The authors suggested that increased metabolic rate may be reflected in the higher hypoxanthine levels. However, lower lactate levels suggest lower production or clearance from the working muscle. Given that hypoxanthine was higher, and this may reflect higher ATP breakdown, this suggests that lactate/H+ levels are likely lower due to increases in clearance, rather than decreases in production, although this is largely unknown from these data alone. However, the lower lactate levels may suggest decreases in fatigue as H+ is known to inhibit metabolic enzymes and cross-bridge formation in skeletal muscle [51]. Supporting this further, YHM is known to alter hemodynamics and increase blood flow to skeletal muscle [7,11]. Therefore, increases in skeletal muscle blood flood from YHM may result in greater removal of fatigue-inducing metabolites despite greater energy breakdown.

For psychometric outcomes, YHM resulted in improvements in feelings of energy, alertness, and fatigue. This was similarly described in resistance-trained males who ingested similar doses of YHM [8]. Taken together, this suggests increases in psychological arousal. The authors suggested that YHM ingestion may increase neural signaling in the pre-frontal cortex, which could lead to more anticipatory neural drive [52]. Furthermore, increases in catecholamines would perpetuate a sympathetic response which would result in greater stimulation and neural activation. Admittedly, the psychological underpinnings of YHM ingestion are grossly understudied and warrant further investigation to understand the underlying mechanisms. 

### 4.2. Anaerobic

The effects of YHM ingestion on anaerobic exercise performance have yielded mixed results [3,8,16]. Ostojic et al. conducted a chronic supplementation trial in male professional soccer players where participants consumed either 20 mg/day of YHM or a placebo for 21 days [3]. Metrics of body composition, upper/lower body strength, sprinting power, and agility were obtained before and after the supplement period. While fat mass and % body fat decreased with YHM supplementation, no differences in performance metrics for strength, sprinting power, or agility were observed. While not confirmed in the study directly, authors postulated that body fat changes were likely an effect of catecholamine-induced lipolysis and possibly satiety. The reasons for the lack of changes in performance are not fully clear, but what is worth noting is that, to date, optimal dosing strategies and sports-specific applications remain unknown. Barnes et al. investigated acute YHM supplementation versus placebo on sprint performance in trained females [16]. Participants ingested a single dose of 2.5 mg of YHM 20 min prior to completing 3 × 15 s maximal cycling sprints. YHM supplementation resulted in higher mean power output and lower fatigue index over the sprints. Physiologically, YHM ingestion resulted in greater plasma catecholamines, higher heart rate, and lower blood lactate levels. This suggests that acute YHM ingestion likely initiates a stimulative response, as evidenced by increased power output/maintenance and plasma catecholamines, but may also alter blood flow to active skeletal muscle. As previously mentioned, YHM has been established as a modulator of peripheral blood flow [38,53,54]. Lower post-exercise lactate and fatigue index values may indicate greater lactate clearance, which supports previous findings in aerobic exercise performance following YHM ingestion [14]. Thus, acute YHM ingestion appears to be beneficial for repeated high-intensity or sprint exercise. Recently, Williams et al. investigated the effects of acute YHM versus placebo ingestion on bench press performance in resistance-trained males [8]. Participants ingested 5 mg of YHM or placebo 20 min prior to completing a series of bench press tests. To obtain metrics on explosive ability, participants completed two bench press repetitions @ 75% of 1-RM with maximum explosive intent followed by three sets of repetitions to failure (RTF). Psychological metrics such as feelings of energy, alertness, and motivation were also measured. Velocity and explosive metrics remained unaltered by YHM ingestion. But participants accumulated a greater number of bench press repetitions during the three sets of RTF where improvements were most pronounced in the last set. Feelings of motivation, energy, and alertness were also increased with YHM treatment. Lack of changes in explosive metrics and improvements in repetition volume further support the notion that YHM may be most beneficial in repeated or fatiguing anaerobic exercise. While no blood metrics were analyzed in this study, the accumulation of greater repletion volume may bolster previous findings of lower blood lactate values with YHM as presumably, lower lactate levels may be indicative of lower fatigue, thus allowing for enhanced exercise capacity. However, the improvements in motivation and alertness may have mediated performance through increased effort and optimization of arousal. YHM may be an effective strategy for acutely improving resistance exercise performance, specifically regarding repetition volume and psychological mediators of performance.

While the reason for disparities in performance enhancement findings is not fully clear, it is likely that differences in supplement regimen and time course are mediating differences. Chronic consumption of YHM likely results in desensitization and habituation that may not be experienced with acute dosing regimens. Thus, it is plausible that the ergogenic effects of YHM are transient in nature with optimal benefits being realized through acute supplementation only. Higher and more frequent dosages may accelerate this process, but further research using varying dosages with serial timepoint performance measurements is needed to be conclusive. Additional investigations with larger sample sizes will be needed prior to making widespread recommendations to athletes and recreational exercisers about the ideal ways to supplement with YHM. Furthermore, there have been variable reports on the bioavailability of YHM and individuals may react differently to oral supplementation. Thus, while the evidence supporting low doses for anaerobic performance enhancement is mounting, dose–response research is necessary before safely making widespread recommendations based on the current data available.

## 5. Considerations for Safety and Usage

It is important to note that there are currently no formal guidelines for the dosage or safety of YHM. The U.S. Food and Drug Administration (FDA) previously approved YHM for treating impotence but has since removed this distinction with the identification of therapies with fewer potential side effects [55]. YHM is largely distributed as an unregulated dietary supplement, leading to questionable quality and safety standards in some commercial products [1,6]. Data from poison control and food safety centers internationally have shown a notable increase in reported adverse events associated with YHM-containing products over the past two decades, underscoring the importance of cautious use, especially in individuals with pre-existing cardiovascular or neurological conditions [55,56]. Additionally, YHM sourced directly from yohimbe bark carries additional risks due to its variable alkaloid concentrations, which complicate dosage accuracy and may increase the likelihood of side effects [57]. Indeed, concentrations of YHM in bark extracts are highly variable and can range from 0.2 to 15% of total weight, which has led to regulatory agencies, such as the European Safety Authority (EFSA), whose position remains that insufficient evidence is available to provide advice for daily intake [55]. Therefore, supplementing from raw bark is discouraged and standardized forms are preferable for controlled dosing and more predictable pharmacological effects [55]. However, many European countries have banned bark extracts containing YHM due to concerns about purity and poor regulation. For medicinal purposes of impotence and hypotension, maximum doses have been suggested at 30 mg/day, although YHM is rarely used to treat medical conditions anymore due to the availability of treatment with higher specificity and regulation [55]. However, dosing recommendations for exercise performance have yet to be established, especially in the context of safety. Disparities in purity and safety in consumer products suggest that caution should be used when acquiring YHM in commercially available forms. Furthermore, individuals using YHM should check with local law and regulations to ensure that use is permitted, which could be of concern since YHM is widely available online.

Although comprehensive placebo-controlled studies on safety in humans are very limited, acute YHM ingestion at low doses appears (2.5–5 mg) to be well tolerated [3,8,14,15,16]. Adverse events reported in the literature have occurred when YHM is consumed in large quantities or combined with other stimulants (e.g., caffeine, synephrine, rauwolscine), exacerbating its sympathomimetic actions [58,59,60,61]. YHM has a well-documented dose-dependent risk profile, with higher doses linked to gastrointestinal distress, hypertension, tachycardia, anxiety, sweating, and headaches, largely attributed to its central adrenergic activity [25]. In rare cases, severe reactions such as seizures, loss of consciousness, and even death have been reported [58,61]. However, these adverse events have rarely been seen with low doses of YHM, and due to its short half-life, most adverse effects typically resolve on their own without medical intervention [25,57,62]. Existing studies on YHM supplementation for exercise performance have generally reported no adverse effects, suggesting it may be safe within controlled limits [3,8,14,15,16,17,57]. Nonetheless, further research is essential to thoroughly evaluate YHM’s safety profile during physical exertion and individuals should use temperance if beginning a YHM dosing regimen. 

## 6. Conclusions

A challenge in making recommendations supporting the use of YHM currently is the lack of regulation and oversight of purity, form, contamination, etc. It is also not well known how YHM may influence the action of other supplements in multi-ingredient formulas that are commonly sold commercially. While the current review focused on studies exclusively using the isolated compound, the future study of standardized multi-ingredient supplements is a dire need since inclusion in these formulas is the most widely available on the consumer market. Furthermore, optimal dosing has not been established, so it is currently unknown if lower or higher doses could be more effective with minimal side effects. At this time, YHM is not banned from sports competitions and is not on the World Anti-doping Agency’s prohibited substance list. However, YHM alters heart rate variability (HRV), a commonly used metric for monitoring recovery in athletes. Alterations in HRV from YHM may make alter recovery and result in monitoring inaccuracies. Future research on chronic supplementation and possible changes in exercise recovery is needed before recommending long-term use.

Overall, YHM appears to be an effective ergogenic aid for endurance and anaerobic exercise. The mediators of this are through a myriad of physiological and psychological changes including lower lactate/fatigue, greater oxygen uptake, improved motivation, and increased alertness. YHM is a potent sympathomimetic and serious side effects have been reported for higher doses and should likely be avoided by individuals with pre-existing health conditions at this time. Individuals using YHM in attempts to boost exercise performance should use temperance as interindividual differences in pharmacokinetics have been reported. However, lower doses (2.5–5 mg), which have been shown to enhance performance, appear to be well tolerated in healthy populations and may be effective at acutely improving exercise performance.

## Data Availability

Not applicable.

## References

[1] Tam S.W., Worcel M., Wyllie M. (2001). Yohimbine: A clinical review. Pharmacol. Ther..

[2] Teloken C., Rhoden E.L., Sogari P., Dambros M., Vargas Souto C.A. (1998). Therapeutic effects of high dose yohimbine hydrochloride on organic erectile dysfunction. J. Urol..

[3] Ostojic S.M. (2006). Yohimbine: The effects on body composition and exercise performance in soccer players. Res. Sports Med..

[4] Morales A. (2000). Yohimbine in erectile dysfunction: The facts. Int. J. Impot. Res..

[5] Kotanska M., Marcinkowska M., Knutelska J., Zygmunt M., Sapa J. (2018). Yohimbine improves lipid and carbohydrate profiles without reduction in body weight in obese leptin-deficient ob/ob mice. J. Pre-Clin. Clin. Res..

[6] Cohen P.A., Wang Y.H., Maller G., DeSouza R., Khan I.A. (2016). Pharmaceutical quantities of yohimbine found in dietary supplements in the USA. Drug Test. Anal..

[7] Cameron O.G., Zubieta J.K., Grunhaus L., Minoshima S. (2000). Effects of yohimbine on cerebral blood flow, symptoms, and physiological functions in humans. Psychosom. Med..

[8] Williams T.D., Boag L.E., Helton C.L., Middleton M.L., Rogers R.R., Sternenberg L.H., Ballmann C.G. (2022). Effects of Acute Yohimbine Hydrochloride Ingestion on Bench Press Performance in Resistance-Trained Males. Muscles.

[9] Goldberg M.R., Hollister A.S., Robertson D. (1983). Influence of yohimbine on blood pressure, autonomic reflexes, and plasma catecholamines in humans. Hypertension.

[10] Murburg M.M., Villacres E.C., Ko G.N., Veith R.C. (1991). Effects of yohimbine on human sympathetic nervous system function. J. Clin. Endocrinol. Metab..

[11] Grossman E., Rea R.F., Hoffman A., Goldstein D.S. (1991). Yohimbine increases sympathetic nerve activity and norepinephrine spillover in normal volunteers. Am. J. Physiol. Regul. Integr. Comp. Physiol..

[12] McCarty M.F. (2002). Pre-exercise administration of yohimbine may enhance the efficacy of exercise training as a fat loss strategy by boosting lipolysis. Med. Hypotheses.

[13] Anderson C., Anderson D., Harre N., Wade N. (2013). Case study: Two fatal case reports of acute yohimbine intoxication. J. Anal. Toxicol..

[14] Ballmann C.G., Rogers R.R., Barnes M.E., Cowan C.R., Elwell C.C., Luiken K.A., Lehman G.Y., Kaylor J.C., Simpson E.G., Westbrooks S.B. (2024). Yohimbine Ingestion Mitigates Morning-Associated Decrements in High-Intensity Exercise Performance. Nutraceuticals.

[15] M Al-Kuraishy H., AN Abood H., Al-Gareeb I.A. (2014). Ergogenic Effects of Yohimbine: Standardized Cycling Clinical Study. Kerbala J. Med..

[16] Barnes M.E., Cowan C.R., Boag L.E., Hill J.G., Jones M.L., Nixon K.M., Parker M.G., Parker S.K., Raymond M.V., Sternenberg L.H. (2022). Effects of Acute Yohimbine Hydrochloride Supplementation on Repeated Supramaximal Sprint Performance. Int. J. Environ. Res. Public Health.

[17] Barnes M.E., Williams T., Cowan C.R., Torres B.A., Clark W.T., Rogers R.R., Harms L.R., Ballmann C.G. (2023). The Effects of Acute Rauwolscine (α-Yohimbine) Ingestion on Repeated Wingate Sprint Performance in Healthy Males. Top. Exerc. Sci. Kinesiol..

[18] Hai-Bo L., Yong P., Lu-qi H., Jun X., Pei-Gen X. (2013). Mechanism of selective inhibition of yohimbine and its derivatives in adrenoceptor α2 subtypes. J. Chem..

[19] Giovannitti J.A., Thoms S.M., Crawford J.J. (2015). Alpha-2 adrenergic receptor agonists: A review of current clinical applications. Anesth. Prog..

[20] Koganei H., Takeuchi A., Kimura T., Satoh S. (1995). Effects of yohimbine and desipramine on adrenal catecholamine release in response to splanchnic nerve stimulation in anesthetized dogs. Biol. Pharm. Bull..

[21] Waluga, Janusz, Karpel, Hartleb, Nowak (1998). Cardiovascular effects of ephedrine, caffeine and yohimbine measured by thoracic electrical bioimpedance in obese women. Clin. Physiol..

[22] Owen J., Nakatsu S., Fenemore J., Condra M., Surridge D., Morales A. (1987). The pharmacokinetics of yohimbine in man. Eur. J. Clin. Pharmacol..

[23] Freitas F., Nascimento N., Cerqueira J., Morais M., Regadas R., Gonzaga-Silva L. (2009). Yohimbine relaxes the human corpus cavernosum through a non-adrenergic mechanism involving the activation of K+ ATP-dependent channels. Int. J. Impot. Res..

[24] McMahon S., Jenkins D. (2002). Factors affecting the rate of phosphocreatine resynthesis following intense exercise. Sports Med..

[25] Jabir N.R., Firoz C.K., Zughaibi T.A., Alsaadi M.A., Abuzenadah A.M., Al-Asmari A.I., Alsaieedi A., Ahmed B.A., Ramu A.K., Tabrez S. (2022). A literature perspective on the pharmacological applications of yohimbine. Ann. Med..

[26] Galitzky J., Taouis M., Berlan M., Riviere D., Garrigues M., Lafontan M. (1988). α2-Antagonist compounds and lipid mobilization: Evidence for a lipid mobilizing effect of oral yohimbine in healthy male volunteers. Eur. J. Clin. Investig..

[27] Herman A.M., Critchley H.D., Duka T. (2019). The impact of Yohimbine-induced arousal on facets of behavioural impulsivity. Psychopharmacology.

[28] Huang M., Messing R.B., Sparber S.B. (1987). Learning enhancement and behavioral arousal induced by yohimbine. Life Sci..

[29] Swann A.C., Lijffijt M., Lane S.D., Cox B., Steinberg J.L., Moeller F.G. (2013). Norepinephrine and impulsivity: Effects of acute yohimbine. Psychopharmacology.

[30] Le Corre P., Dollo G., Chevanne F., Le Verge R. (1999). Biopharmaceutics and metabolism of yohimbine in humans. Eur. J. Pharm. Sci..

[31] Millan M.J., Newman-Tancredi A., Audinot V., Cussac D., Lejeune F., Nicolas J.P., Cogé F., Galizzi J.P., Boutin J.A., Rivet J.M. (2000). Agonist and antagonist actions of yohimbine as compared to fluparoxan at α2-adrenergic receptors (AR) s, serotonin (5-HT) 1A, 5-HT1B, 5-HT1D and dopamine D2 and D3 receptors. Significance for the modulation of frontocortical monoaminergic transmission and depressive states. Synapse.

[32] Hedner T., Edgar B., Edvinsson L., Hedner J., Persson B., Pettersson A. (1992). Yohimbine pharmacokinetics and interaction with the sympathetic nervous system in normal volunteers. Eur. J. Clin. Pharmacol..

[33] Hoffman J.R., Kang J., Ratamess N.A., Hoffman M.W., Tranchina C.P., Faigenbaum A.D. (2009). Examination of a pre-exercise, high energy supplement on exercise performance. J. Int. Soc. Sports Nutr..

[34] Hoffman J.R., Kang J., Ratamess N.A., Rashti S.L., Faigenbaum A.D. (2008). Thermogenic effect of a high energy, pre-exercise supplement. Kinesiology.

[35] Lutsch D.J., Camic C.L., Jagim A.R., Stefan R.R., Cox B.J., Tauber R.N., Henert S.E. (2020). Effects of a multi-ingredient preworkout supplement versus caffeine on energy expenditure and feelings of fatigue during low-intensity treadmill exercise in college-aged males. Sports.

[36] French D.N., Kraemer W.J., Volek J.S., Spiering B.A., Judelson D.A., Hoffman J.R., Maresh C.M. (2007). Anticipatory responses of catecholamines on muscle force production. J. Appl. Physiol..

[37] Tank J., Heusser K., Diedrich A., Brychta R.J., Luft F.C., Jordan J. (2007). Yohimbine attenuates baroreflex-mediated bradycardia in humans. Hypertension.

[38] Kenney W., Zappe D., Tankersley C., Derr J. (1994). Effect of systemic yohimbine on the control of skin blood flow during local heating and dynamic exercise. Am. J. Physiol.-Heart Circ. Physiol..

[39] Gomez-Ambrosi J., Fruhbeck G., Aguado M., Milagro F.I., Margareto J., Martinez A.J. (2001). Divergent effects of an α2-adrenergic antagonist on lipolysis and thermogenesis: Interactions with a β3-adrenergic agonist in rats. Int. J. Mol. Med..

[40] Lafontan M., Berlan M., Galitzky J., Montastruc J.-L. (1992). Alpha-2 adrenoceptors in lipolysis: α2 antagonists and lipid-mobilizing strategies. Am. J. Clin. Nutr..

[41] Kim J., Park J., Lim K. (2016). Nutrition supplements to stimulate lipolysis: A review in relation to endurance exercise capacity. J. Nutr. Sci. Vitaminol..

[42] Uth N., Sørensen H., Overgaard K., Pedersen P.K. (2004). Estimation of
$\dot{V}$O_2max_ from the ratio between HR_max_ and HR_rest_–the Heart Rate Ratio Method. Eur. J. Appl. Physiol..

[43] Mahon A.D., Stephens B.R., Cole A.S. (2008). Exercise responses in boys with attention deficit/hyperactivity disorder: Effects of stimulant medication. J. Atten. Disord..

[44] Toner M.M., Kirkendall D., Delio D., Chase J., Cleary P., Fox E. (1982). Metabolic and cardiovascular responses to exercise with caffeine. Ergonomics.

[45] Bell D.G., Jacobs I., Zamecnik J. (1998). Effects of caffeine, ephedrine and their combination on time to exhaustion during high-intensity exercise. Eur. J. Appl. Physiol. Occup. Physiol..

[46] Drust B., Waterhouse J., Atkinson G., Edwards B., Reilly T. (2005). Circadian rhythms in sports performance—An update. Chronobiol. Int..

[47] Blazer H.J., Jordan C.L., Pederson J.A., Rogers R.R., Williams T.D., Marshall M.R., Ballmann C.G. (2021). Effects of time-of-day training preference on resistance-exercise performance. Res. Q. Exerc. Sport.

[48] Dumar A.M., Huntington A.F., Rogers R.R., Kopec T.J., Williams T.D., Ballmann C.G. (2021). Acute beetroot juice supplementation attenuates morning-associated decrements in supramaximal exercise performance in trained sprinters. Int. J. Environ. Res. Public Health.

[49] Roatta S., Arendt-Nielsen L., Farina D. (2008). Sympathetic-induced changes in discharge rate and spike-triggered average twitch torque of low-threshold motor units in humans. J. Physiol..

[50] Roatta S., Farina D. (2010). Sympathetic actions on the skeletal muscle. Exerc. Sport Sci. Rev..

[51] Fitts R.H. (2008). The cross-bridge cycle and skeletal muscle fatigue. J. Appl. Physiol..

[52] Sun H., Green T.A., Theobald D.E., Birnbaum S.G., Graham D.L., Zeeb F.D., Nestler E.J., Winstanley C.A. (2010). Yohimbine increases impulsivity through activation of cAMP response element binding in the orbitofrontal cortex. Biol. Psychiatry.

[53] Farrow S., Mers A., Banta G., Steigerwalt S., Lockette W. (1990). Effect of the alpha 2-adrenergic antagonist yohimbine on orthostatic tolerance. Hypertension.

[54] Ernst E., Pittler M. (1998). Yohimbine for erectile dysfunction: A systematic review and meta-analysis of randomized clinical trials. J. Urol..

[55] EFSA Panel on Food Additives and Nutrient Sources Added to Food (ANS) (2013). Scientific Opinion on the evaluation of the safety in use of Yohimbe (Pausinystalia yohimbe (K. Schum.) Pierre ex Beille). EFSA J..

[56] Kearney T., Tu N., Haller C. (2010). Adverse drug events associated with yohimbine-containing products: A retrospective review of the California Poison Control System reported cases. Ann. Pharmacother..

[57] Cimolai N. (2017). An overview of yohimbine in sports medicine. Sustained Energy for Enhanced Human Functions and Activity.

[58] Zhu L., Han X., Zhu J., Du L., Liu L., Gong W. (2021). Severe acute intoxication with yohimbine: Four simultaneous poisoning cases. Forensic Sci. Int..

[59] Giampreti A., Lonati D., Locatelli C., Rocchi L., Campailla M.T. (2009). Acute neurotoxicity after yohimbine ingestion by a body builder. Clin. Toxicol..

[60] Bridwell R.E., Yoo M.J., Grove J.J., Ng P.C. (2020). Chest pain from supplement use in an active duty soldier: A case report. Mil. Med..

[61] Drevin G., Palayer M., Compagnon P., Zabet D., Jousset N., Briet M., Abbara C. (2020). A fatal case report of acute yohimbine intoxication. Forensic Toxicol..

[62] Vogt H., Brandl P., Kockott G., Schmitz J., Wiegand M., Schadrack J., Gierend M. (1997). Double-blind, placebo-controlled safety and efficacy trial with yohimbine hydrochloride in the treatment of nonorganic erectile dysfunction. Int. J. Impot. Res..

